# Synthesis and Modifications of Phosphinic Dipeptide Analogues

**DOI:** 10.3390/molecules171113530

**Published:** 2012-11-15

**Authors:** Artur Mucha

**Affiliations:** Department of Bioorganic Chemistry, Faculty of Chemistry, Wrocław University of Technology, Wybrzeże Wyspiańskiego 27, 50-370 Wrocław, Poland; Email: artur.mucha@pwr.wroc.pl; Tel.: +48-71-320-3446; Fax: +48-71-320-2427

**Keywords:** phosphinic peptides, P-C bond formation, multicomponent reactions, side-chain diversification, enzyme inhibition

## Abstract

Pseudopeptides containing the phosphinate moiety (-P(O)(OH)CH_2_-) have been studied extensively, mainly as transition state analogue inhibitors of metalloproteases. The key synthetic aspect of their chemistry is construction of phosphinic dipeptide derivatives bearing appropriate side-chain substituents. Typically, this synthesis involves a multistep preparation of two individual building blocks, which are combined in the final step. As this methodology does not allow simple variation of the side-chain structure, many efforts have been dedicated to the development of alternative approaches. Recent achievements in this field are summarized in this review. Improved methods for the formation of the phosphinic peptide backbone, including stereoselective and multicomponent reactions, are presented. Parallel modifications leading to the structurally diversified substituents are also described. Finally, selected examples of the biomedical applications of the title compounds are given.

## Abbreviations

Aa_1_ψ[P(O)(OH)X]-Aa_2_: phosphorus-containing dipeptide analogues (X = NH, phosphonamidates; X = O, phosphonates; X = CH_2_, phosphinates)
AaPH: α-amino-*H*-phosphinic (phosphonous) acid
Ac: acetyl
Ad: 1-adamantyl
Alk: alkyl
Alloc: allyloxycarbonyl
APN: alanyl aminopeptidase
Ar: aryl
ATP: adenosine triphosphate
Boc: *t*-butyloxycarbonyl
BOP: (benzotriazol-1-yloxy)tris(dimethylamino)phosphonium hexafluorophosphate
BSA: *N,O*-bis(trimethylsilyl)-acetamide
*i*-Bu: isobutyl
*n*-Bu: *n*-butyl
*sec*-Bu: *sec*-butyl
*t*-Bu: *tert*-butyl
Bz: benzoyl
Bzl: benzyl
Cbz: benzyloxy-carbonyl
CPB: carboxypeptidase B
dba: dibenzylideneacetone
DEAD: diethyl azodicarboxylate
DCC: *N,N'*-dicyclohexylcarbodiimide
*de*: diastereomeric excess
DIPEA: diisopropylethylamine
DMAP: 4-dimethylaminopyridine
DME: 1,2-dimethoxy-ethane
DMF: dimethylformamide
EDC: 1-ethyl-3-(3'-dimethylaminopropyl)carbo-diimide
Et: ethyl
EWG: electron withdrawing group
Fmoc: 9-fluorenylmethoxy-carbonyl
HMDS: 1,1,1,3,3,3-hexamethyldisilazane
HOBt: 1-hydroxybenzotriazole
HPLC: high performance liquid chromatography
LAP: leucine aminopeptidase
Me: methyl
MurD: UDP-*N*-acetylmuramoyl-L-alanyl:D-glutamate ligase
Nu: nucleophile
Pac: phenacyl
PG: protecting group
Ph: phenyl
Phth: phthaloyl
*i*-Pr: isopropyl
PyBOP: (benzotriazol-1-yloxy)tris(pyrrolidino)phosphonium hexafluorophosphate
SPPS: solid-phase peptide synthesis
Su: succinimide
TAFIa: thrombin-activatable fibrinolysis inhibitor
TFA: trifluoroacetic acid
THF: tetrahydrofuran
Tl: triflate (trifluoromethane- sulfonate)
TLC: thin layer chromatography
TMSCl: chlorotrimethyl-silane
Tr: trityl (triphenylmethyl)
TS: transition state
Ts: tosyl
Trs: 2,4,6-triisopropylphenylsulfonyl

## 1. Introduction

**Figure 1:**
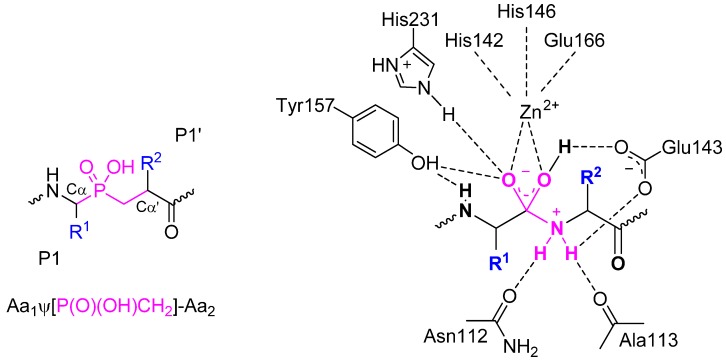
A general structure for phosphinic dipeptide analogues and the resemblance of this structure to the tetrahedral transition state of peptide hydrolysis postulated for thermolysin [1]. Side chains R^1^ and R^2^ are specific to the S1 and S1' binding pocket and are also referred to as the P1 and P1' substituents, according to the nomenclature proposed by Schechter and Berger [2].

Phosphinic pseudodipeptides (Figure 1) are typically defined as dipeptide analogues that replace the amide bond with the phosphinate moiety. The rationale behind the replacement is not to mimic the peptide linkage alone, explicitly in its ground state, but the assumed analogy concerns isosteric and isoelectronic resemblances to the high-energy tetrahedral transition state (TS) of the amide hydrolysis. 

Translation of the TS arrangement into the inhibitor structure is one of the fundamental concepts in drug design, originating directly from the early suggestion of evolutionary orientation of the enzyme active sites to bind substrates optimally in this state, which diminishes the energy of activation of the catalyzed reaction [3]. Formally, the binding of TS inhibitors should be tighter than the binding of the substrates by the factor of the enzymatic rate enhancement [4].

Three different types of phosphorus-containing peptide analogues, which comprise the structural fragment Aa_1_ψ[P(O)(OH)X]-Aa_2_, have been constructed to follow this idea. The phosphorus-containing peptide analogues include: phosphonamidates (X = NH, the closest TS analogues), phosphonates (X = O, pseudodepsipeptides) and phosphinates (X = CH_2_). All these compounds appear particularly effective in regulating the activity of metalloproteases. Nevertheless, investigation of other proteases (e.g., aspartyl) and, in general, other classes of enzymes (e.g., ligases) also brought inhibition to an impressive level [5,6,7]. In the early period of these studies (during the 80s of the 20th century), phosphonamidate and phosphonate pseudopeptides attracted major attention. These pseudopeptides emerged as invaluable tools in fundamental structural and mechanistic studies carried out on prototypical metalloproteases, thermolysin and carboxypeptidase A [1,8,9,10,11,12,13,14]. Certain drawbacks, such as hydrolytic instability of the P-N bond and frequent limited activity of the P-O derivatives excluded them however from later practical applications.

Phosphinic compounds avoid these inconveniences. Phosphinates are stable over the whole pH range and equipotent with or only slightly less potent than the corresponding phosphonamidate species [15]. The utility potential of phosphinic peptides in drug design was greatly increased by development of a synthetic procedure leading to the Fmoc-Aa_1_ψ[P(O)(OAd)CH_2_]-Aa_2_ building block [16]. This synthon is compatible with standard methodologies of solid-phase peptide synthesis (SPPS) and combinatorial synthesis. Since then, numerous active sequences have been obtained, and this progress is relevantly and comprehensively reviewed elsewhere [5,6,7,17,18,19,20,7,17]. The phosphinate chemistry was also historically systematized in an excellent paper by Yiotakis *et al.* in 2004 [21]. Despite considerable achievements, construction of a fundamental phosphinic α,α'-dipeptide, which comprises appropriate side-chains remains the main challenge in the field. The current review is dedicated entirely to this synthetic aspect with particular attention to diversification of the P1 and P1' substituents. The contents are limited to recent achievements. In principle, the literature data published after 2000 are discussed, and earlier papers are only selectively highlighted to give the proper background.

## 2. Synthesis of the Phosphinic α,α'-Dipeptide Backbone

### 2.1. Retrosynthetic Analysis

**Scheme 1:**
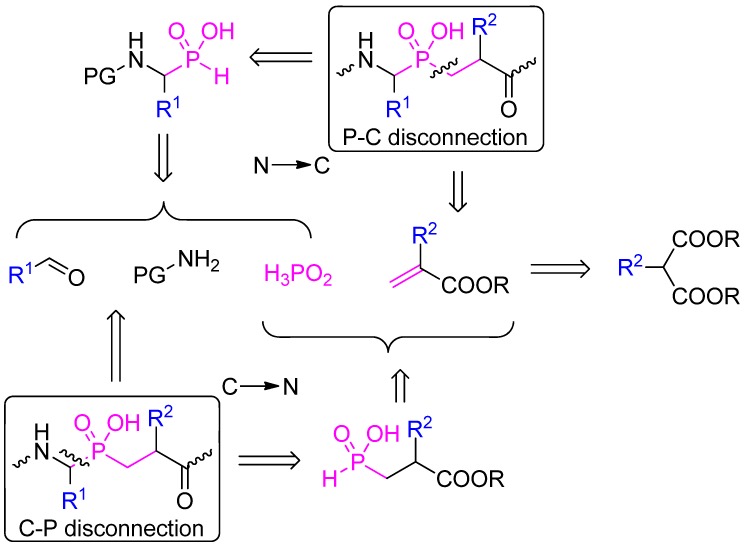
Retrosynthetic analysis of the phosphinic dipeptide scaffold.

Approaches based on a phospha-Michael addition to obtain the C-P-C pseudodipeptide skeleton predominate. The most traditional approach involves the addition of a suitably *N*-protected α-amino-*H*-phosphinic (phosphonous, AaPH) acid or its ester (alkyl *H*-phosphinate) to an acrylate (P-C disconnection, synthetic direction from the N to C terminus, Scheme 1). The *H*-phosphinic acid component requires activation to the more nucleophilic tervalent ester form that is typically achieved in the presence of a silylation agent. On the other hand, the reaction of alkyl *H*-phosphinate esters can also be catalyzed with a strong base. α-Amino-*H*-phosphinic substrates are conveniently available from appropriate aldehydes, amino components and hypophosphorous acid. Acrylates (α-substituted α,β-unsaturated esters) also need to be synthesized separately, typically by a Knoevenagel reaction of α-substituted malonate monoesters and formaldehyde.

Alternatively, a Michael-type addition can precede incorporation of the *N*-terminal fragment. The essential substrate, β-substituted β-alkoxycarbonylethyl-*H*-phosphinic acid, is readily obtained in the addition of bis(trimethylsilyl) phosphonite to an acrylate, similarly to the reactions previously mentioned. The product is then subjected to a three-component condensation with an aldehyde and amino/amido component (C-P disconnection, C → N, Scheme 1). Depending on the exact structures of the reactants, this reaction is a variant of the Kabachnik-Fields, phospha-Mannich or amidoalkylation reaction.

### 2.2. N → C Strategy

**Scheme 2:**
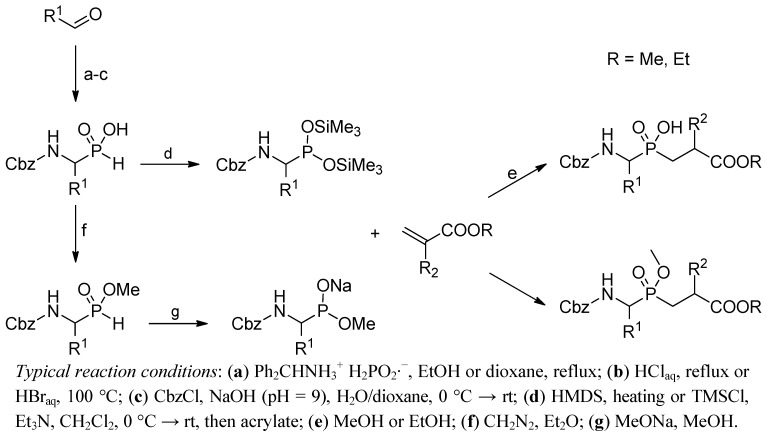
A general overview of the synthetic strategies leading to phosphinic dipeptide analogues by addition of α-amino-*H*-phosphinates (acids or esters) to acrylates.

Historically, addition of α-amino-*H*-phosphinates (acids and esters) to acrylates has been the most commonly recognized approach to construct phosphinic dipeptide analogues (Scheme 2). This popularity has been stimulated by an easy access to suitably protected phosphorus substrates. Different variations for obtaining these P-H substrates, suggested originally by Baylis *et al.*, are based mainly on a three-component condensation of an aldehyde, hypophosphorous acid and diphenylmethylamine as the nitrogen source, or addition of H_3_PO_2_ to diphenylmethylimines [22]. The diphenylmethyl group can be hydrolyzed from the adduct under acidic conditions to obtain free α-aminophosphonous acids. The main advantages of the approach are simplicity and low cost, even though the preparative yield is not high. The amino group can subsequently be protected by the benzyloxycarbonyl group (Cbz) in a water solution [22]. The procedure is particularly convenient for alkyl and aryl substituents with a single and unpredictable exception of GlyPH. An additional side-chain functionality needs attention with respect to its orthogonal protection during the synthesis of α-aminophosphonous acids and their further transformations.

Benzyloxycarbonyl is a standard group used for N-protection, but other carbamates are compatible with the phospha-Michael addition conditions, particularly with the mild TMSCl/tertiary amine version. These other carbomates commonly include *t*-butyloxycarbonyl (Boc) [23] and 9-fluorenyl- methoxycarbonyl (Fmoc) [24]. Other specific protecting groups will be also mentioned in the text.

The *H*-phosphinic component requires activation to the tervalent nucleophilic ester form, typically accomplished by silylation (Scheme 2). Alternatively, the effect can be achieved by heating with hexamethyldisilazane (HMDS), by action of *N,O*-bis(trimethylsilyl)acetamide (BSA), or in the presence of chlorotrimethylsilane (TMSCl) and a tertiary amine [21,25,26,27,28,29,30]. After addition to an acrylate, the silylated adduct is decomposed with an alcohol or water.

Transformation of *N*-protected α-amino-*H*-phosphinic acid into its ester provides another opportunity for activation. This reaction involves the action of a strong base, e.g., sodium methoxide or potassium *t*-butoxide, to shift the equilibrium from the P(V) tautomer to a more nucleophilic P(III) species (Scheme 2) [21,31,32,33,34].

As mentioned above, this method for obtaining a phosphinic dipeptide backbone is not problematic for alkyl, aryl or alkylaryl P1 residues that do not contain an additional functionality, and accordingly, numerous structures have become available in this manner [16,21,35,36]. However, more complex Cα side-chains that incorporate a certain heteroatom moiety can greatly influence the chemical behavior of the phosphorus substrate. For example, the neighboring assistance of a carboxylate group (free or esterified) in the phosphonous analogue of aspartic acid did not permit the Michael addition to occur [23]. Even though the target phosphinic compound is obtained, further transformations of this phosphinic compound are frequently altered by the complexity of the protection/deprotection strategy. Cbz-Lys(Phth)ψ[P(O)(OH)CH_2_]-Ala-OMe can serve as a representative example [37]. The attempts to prepare its free carboxylate form (with all other three functionalities blocked), suitable for peptide coupling, failed. In this case, the issue had to be addressed by the use of alternative synthetic approaches.

**Scheme 3:**
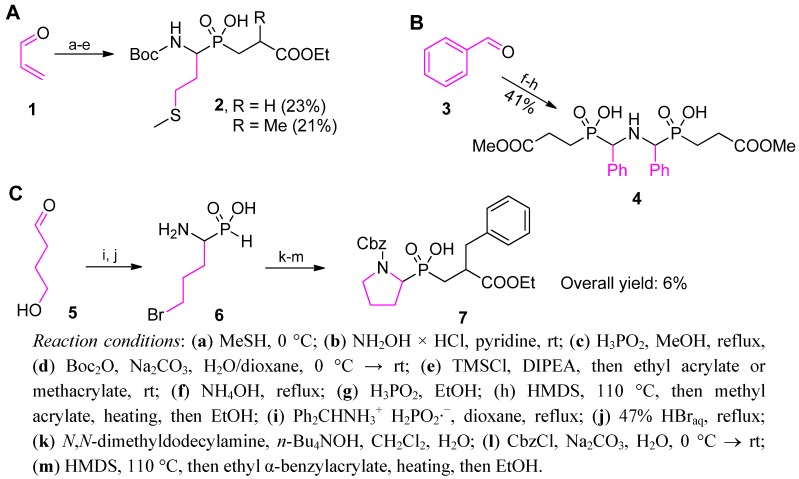
Selected examples of preparation of additionally functionalized α-amino-*H*-phosphinic acids and their application in addition reactions to acrylates.

Selected rare examples of phosphinic dipeptide structures that have been prepared by incorporation of an additionally functionalized α-amino-*H*-phosphinic acid to an acrylate are listed in Scheme 3. The first illustration addresses the preparation of P1 methionine derivatives as inhibitors of metalloproteases [36,38]. To present a less popular version of obtaining the P-H substrate, Liboska *et al.* added methanethiol to acrolein (**1**) and converted the product into the corresponding oxime, which, in turn, reacted with anhydrous hypophosphorous acid (Scheme 3A) [38]. Boc-protected *H*-phosphinic acid was added to acrylates to yield **2** under typical conditions. The second example (Scheme 3B) shows a molecule of *C*_2_ symmetry obtained by Kabudin and Saadati from benzaldehyde (**3**). The final modification to aminobisphosphinic acid **4** was performed in a standard way, however, without any protection of the phosphorus component [39]. Scheme 3C presents the construction of a pseudodipeptide containing a 5-membered ring of proline (**7**) starting from hydroxyaldehyde **5** [40]. To perform this transformation, the Michael addition followed cyclative alkylation of α-amino-ω-bromobutylphosphonous acid (**6**). The overall yield of the five-step procedure was only 6%, whereas the strategy proceeding in the opposite direction (C → N, compare Scheme 1 and chapter 2.3.1) provided the target molecule in 52% yield in three steps [40].

**Figure 2:**
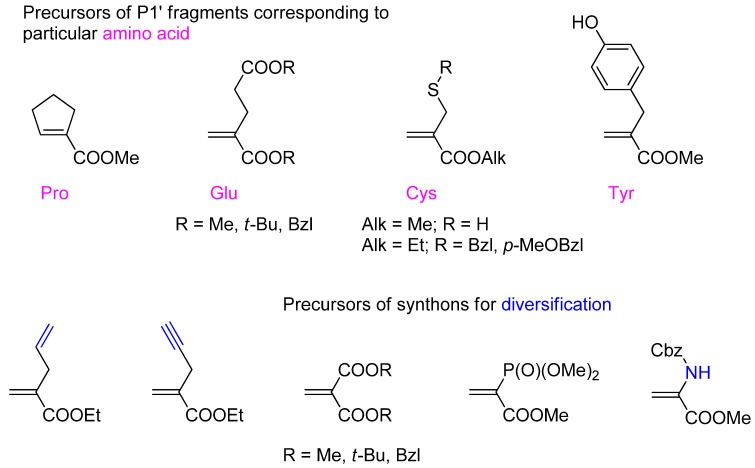
A selection of additionally functionalized α-substituted acrylate derivatives that were found to be compatible with phospha-Michael addition chemistry.

In contrast to the limited tolerance of a P1 functionality, the addition of the P-H moiety to the activated double bond leads to an extensive choice of structurally variable acrylates (Figure 2). Cyclic compounds [41], as well as compounds containing a carboxyl group [42,43,44], a phosphonate [31], a hydroxyl group [35,45], a thiol [31,35] or an amino function [46] in the side chain (typically, suitably protected) and residues of an unsaturated character [47,48] allow an effective reaction. Some of the unsaturated ester substrates are obvious precursors of proteinogenic P1' fragments that ensure specificity of the products in biological studies. Other acrylates are designed to introduce a structural fragment that can be subjected to a further diversification to the phosphinic scaffold.

**Scheme 4:**
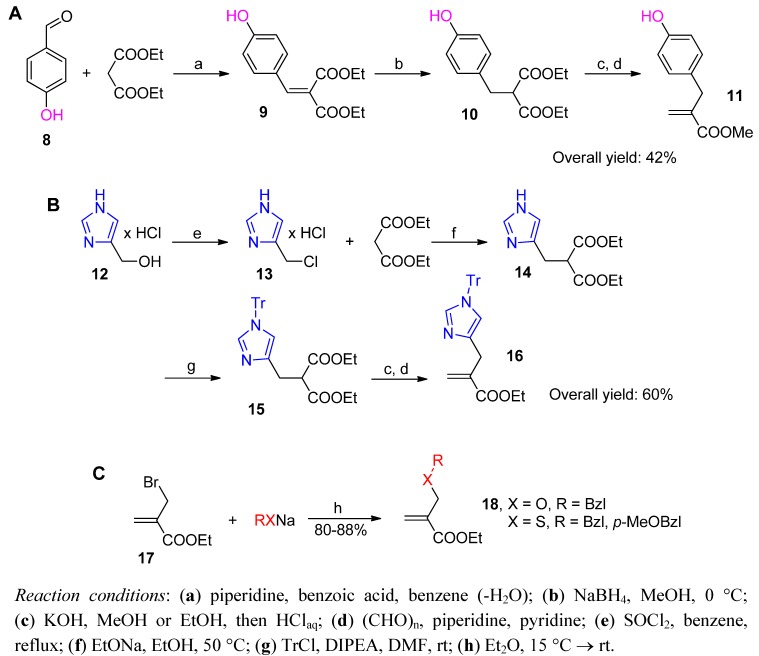
Selected synthetic procedures leading to additionally functionalized α-substituted acrylate derivatives.

Obtaining the acrylates may present individual synthetic challenges (selected examples are shown in Scheme 4). Preparing the appropriate acrylate usually involves a Knoevenagel condensation of appropriately substituted malonate monoester with formaldehyde [49]. The key malonate substrate (e.g., **10**, Scheme 4A) can be prepared by condensation of an aldehyde (such as **8**) with subsequent reduction of the product **9** [45] or by a standard alkylation (e.g., **15**, Scheme 4B) with alkyl halides (such as imidazole derivative **13**) [50]. The total yield of the product (here, **11** and **16**) varies significantly and depends on the structure of the substituent. Other methods have been developed exclusively for particular target derivatives [21,35,36]. For example, β-alkoxy or β-alkylthio derivatives (**18**, Scheme 4C) are prepared efficiently by alkylation of sodium alkoxides or thiolates with α-bromomethyl acrylate [35].

Multistep preparations of the phosphinic dipeptide from acrylates and α-aminophosphonous acids that converge the substrates in the phospha-Michael addition are reproducible and show reasonable yields. Consequently, these preparations have generally been employed for the construction of pseudodipeptides corresponding to both natural and non-natural amino acid sequences. Certain limitations, mostly concerning the P1 substituent structure, were proposed for the multicomponent approaches (see below). Several attempts to modify the original N → C Michael addition have also been undertaken. Most of the attempts at modification involved simplification of the protection/deprotection procedure and merging both P-C bond formation processes in a one-pot manner that reduced the number of the synthetic steps [33,34,51,52]. Typically, an appropriate ester of hypophosphorous acid is treated consecutively with an imine (or triazine) and then by an acrylate. Because these modifications were scarcely noticed in the scientific community, a single and arbitrarily selected case is presented here.

**Scheme 5:**
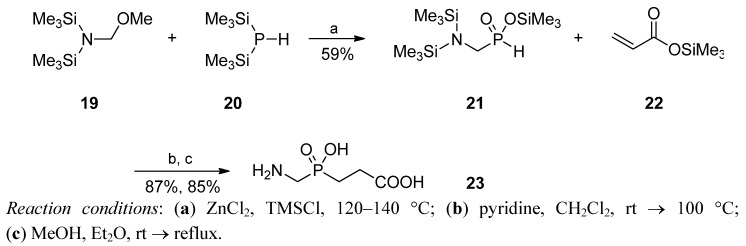
Glyψ[P(O)(OH)CH_2_]-Gly preparation from silylated substrates.

In the course of studies on the reactivity of trimethylsilylated compounds, including (Me_3_SiO)_2_P-H (**20**), Prishchenko *et al.* discovered several reaction variants that led to phosphinic compounds [53,54,55]. Among others, *N*-(methoxymethyl)bis(trimethylsilyl)amine (**19**) reacted with bis(trimethylsilyl) phosphonite in the presence of ZnCl_2_ to yield the GlyPH analogue **21**, which was subsequently added to trimethylsilyl acrylate (**22**) [53]. Free Glyψ[P(O)(OH)CH_2_]-Gly (**23**) was obtained after methanolysis with a total yield of 44% (Scheme 5).

**Scheme 6:**
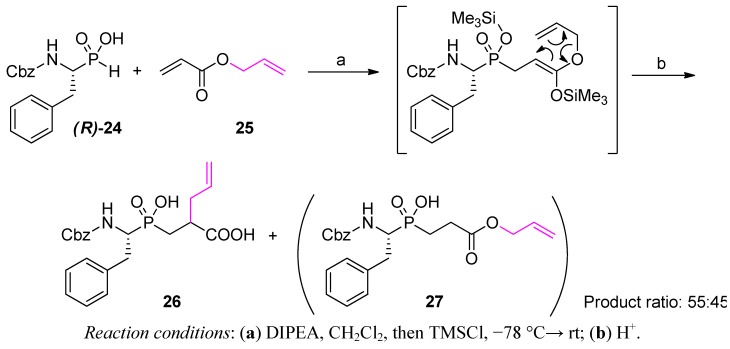
Phospha-Michael addition of α-*N*-benzyloxycarbonylaminoalkyl-*H*-phosphinic acid to allyl acrylate followed by the Ireland-Claisen rearrangement of the product.

An interesting possibility of tandem P-C and C-C bond formation on a phosphinic dipeptide scaffold was recognized in a preliminary way in the Yiotakis group [56]. The tandem bond formation involved phospha-Michael addition of a P-H substrate to allyl acrylate (**25**) followed by a Claisen-type rearrangement (Scheme 6). Unexpectedly, the product of the addition of optically active α-*N*-benzyloxycarbonylaminoalkylphosphonous acid [(***R***)**-24**] rearranged to a lesser extent than structurally less complex phenylphosphinic acid. The ratio of the final product **26** to un-rearranged allyl ester **27** was 55:45, compared to 91:9 for phenylphosphinic acid. An incompletely understood role for the amide N-H in intramolecular proton transfer in the silylated intermediate was suggested as a possible reason. Indeed, bis-N-protection of the α-aminoalkyl-*H*-phosphinic acid substrate increased the preparative yield of the target product from 52% to 84%, comparable to the yield obtained for phenylphosphinic acid. The reaction can be carried out conveniently in a three-component variant using acryloyl chloride (or other α-substituted analogues) and various allyl and propargyl alcohols.

#### Phospha-Michael Addition on Solid Phase

**Scheme 7:**
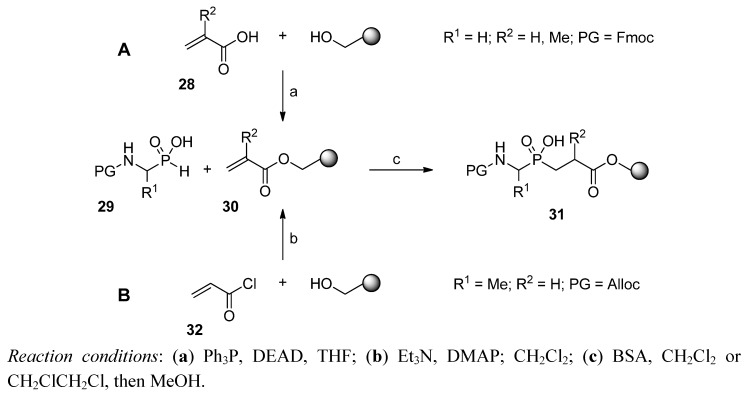
Phospha-Michael addition of N-protected α-aminoalkyl-*H*-phosphinic acid to acrylates immobilized on a solid phase.

Formation of the phosphinic bond could also occur on a solid support. Dorff *et al.* modified Wang resin with acrylic or metacrylic acid (**28**, R^2^ = H or Me) under Mitsunobu conditions (Scheme 7A) [57]. Subsequently, FmocGlyPH (**29**, R^1^ = H, PG = Fmoc), activated by BSA, was added to the immobilized double bond of **30** to give product **31**. After esterification of the phosphinate with diazomethane and Fmoc removal, the N-terminus could easily be modified, e.g., by acyl chlorides or isocyanates [57].

In a similar approach, Buchardt and Meldal acylated a polyethylene glycol/polystyrene resin with acryloyl chloride (**32**), followed by addition of AllocAlaPH (**29**, R^1^ = Me, PG = Alloc, Scheme 7B) [58]. The procedure was then validated in a solid-phase synthesis of an undecapeptide containing an internal Alaψ[P(O)(OH)CH_2_]-Gly unit. Although these methodologies could provide an interesting alternative for the preparation of longer sequences in an automatic manner, the methodologies failed to attract further attention. Introduction of a pre-synthesized pseudodipeptidic building block to the classical SPPS appeared to be appreciated, for the most part.

### 2.3. C → N Strategy

**Scheme 8:**
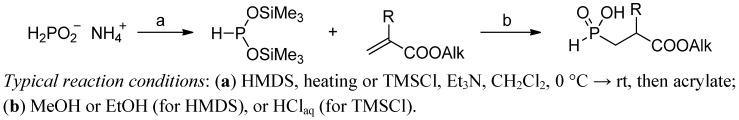
A general scheme for the synthesis of β-substituted β-alkoxycarboxyl-phosphonous acids, precursors of the P1' fragment of phosphinic dipeptides.

Acrylates are also indispensable starting materials in an alternative strategy for phosphinic α,α'-dipeptide synthesis. Similarly to N → C strategy, this approach involves incorporation of structural fragments to the central phosphorus core, but an acrylate, which determines the P1' portion, is added first to the activated silyl ester of hypophosphorous acid (Scheme 8) [29,30]. The reaction, starting from ammonium hypophosphate under the same conditions as described above for α-aminophosphonous acids (see Scheme 2), is carried out and yields a β-substituted β-alkoxycarboxyl-*H*-phosphinic acid. Subsequently, the product is used in a condensation reaction with carbonyl and amine/amide components to build the N-terminal fragment of the molecule.

#### 2.3.1. Amidoalkylation

**Scheme 9:**
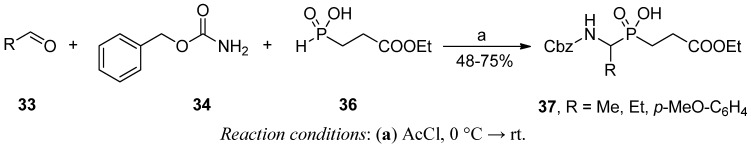
Synthesis of phosphinic dipeptides in a three-component condensation of an aldehyde, benzyl carbamate and 2-ethoxycarbonylethylphosphinic acid.

Obtaining phosphinic pseudodipeptides in a three-component condensation of carbamate, an aldehyde and a β-alkoxycarboxylphosphonous acid was initially suggested by Chen and Coward (Scheme 9) [59].

This reaction was an analogy to the previous applications of less complex phosphorus components (phosphites) as a method to synthesize α-aminophosphonic acid derivatives [60]. Similarly, aliphatic and aromatic aldehydes (**33**, R = Me, Et, *p*-MeO-C_6_H_4_) reacted with benzyl carbamate (**34**) and 2-ethoxycarbonylethylphosphonous acid (**35**) in the presence of acetyl chloride. Conversion proceeded with a good yield (48–75%) to produce the final products **37** after an aqueous workup. The precise role of acetyl chloride as a dehydration (acetylation) solvent/reagent remained ambiguous.

**Scheme 10:**
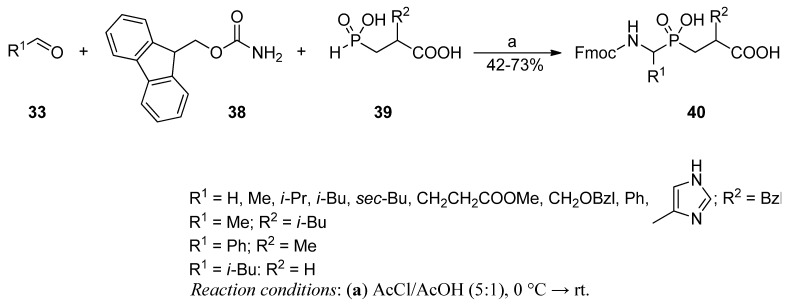
Synthesis of phosphinic dipeptide building blocks suitable for solid phase peptide synthesis in a three-component condensation of an aldehyde, 9-fluorenylmethyl carbamate and 2-substituted 2-carboxyethyl-*H*-phosphinic acid.

Matziari and Yiotakis used the corresponding approach to obtain pseudodipeptides that were directly suitable for solid-phase peptide synthesis [61]. The modification demanded the use of FmocNH_2_ (**38**) instead of CbzNH_2_ and a carboxylic phosphonous acid **39** (R^2^ = H, Me, *i*-Bu, Bzl), instead of an ester to react with different aldehydes **33** in AcCl/AcOH (Scheme 10). *N*-Fmoc protected C-terminal free compounds **40** were isolated with a yield comparable to the yield obtained in the original report of Chen and Coward. The products incorporated P1' residues of glycine, alanine, valine, leucine, isoleucine, phenylglycine and histidine, but also the appropriately protected glutamic acid and serine. Their utility in solid phase preparation of elongated peptide sequences was also confirmed. An EDC/HOBt coupling ensured lack of byproducts despite the presence of a non-protected phosphinate moiety.

**Scheme 11:**
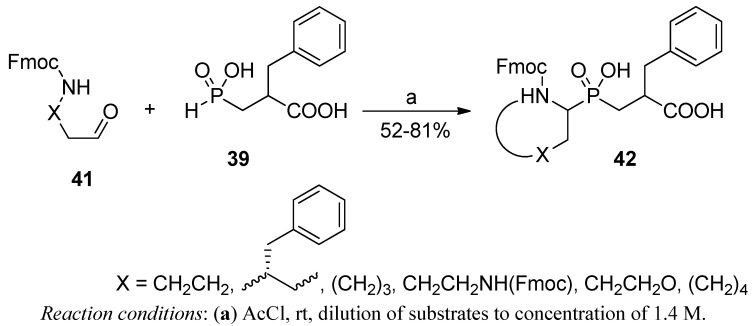
Synthesis of constrained phosphinic dipeptides in an intramolecular three-functional two-component condensation of a 9-fluorenylmethyl carbamate-derived aldehyde and 2-carboxy-3-phenylpropyl-*H*-phosphinic acid.

A similar amidoalkylation reaction performed in an intramolecular manner was employed to obtain P1 constrained peptidomimetics **42** (Scheme 11) [62]. Azacyclic compounds of a 5-, 6- and 7-membered ring were readily synthesized from α,ω-carbamoylaldehydes **41** and phosphinic acid **39** (R = Bzl). Formation of rings of these specific sizes was strongly favored. Attempts to obtain azetidine derivatives were not successful. The cyclization was apparently the driving force for the condensation. No concurrent open-chain products of intermolecular reactions were indicated in competitive experiments.

**Scheme 12:**
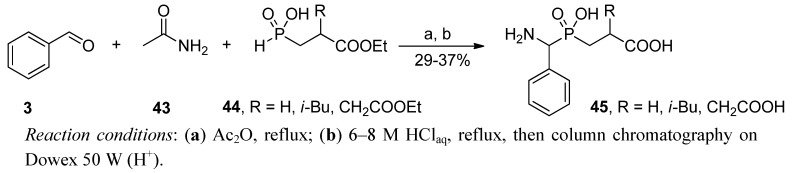
Synthesis of deprotected phosphinic dipeptides in a three-component condensation of benzaldehyde, acetamide, and 2-substituted 2-ethoxycarbonylethyl-*H*-phosphinic acid.

Based on the same general methodology, Rozhko and Ragulin synthesized fully deprotected analogues in a two-step procedure [63]. The condensation of benzaldehyde (**3**), acetamide (**43**), and a phosphonous acid **44** (R = H, *i*-Bu, CH_2_COOEt) in acetic anhydride was simply followed by acid hydrolysis (Scheme 12). Phosphinic compounds **45** were isolated with a low yield (29–37%) using ion exchange chromatography.

**Scheme 13:**
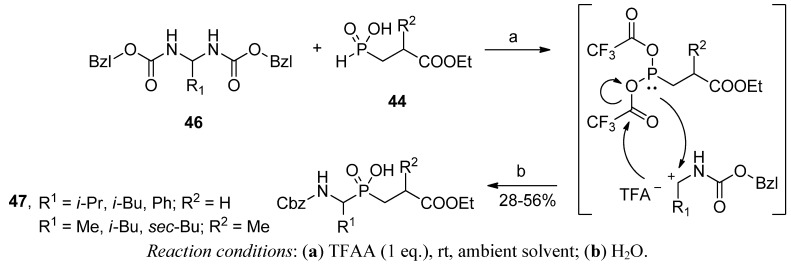
Synthesis of phosphinic dipeptides in the reaction of benzyl biscarbamate and a 2-ethoxycarbonylethyl-*H*-phosphinic acid in the presence of trifluoroacetic anhydride (TFAA) and a suggested mechanism for substrate activation.

As mentioned, the precise role of active carboxyl compounds (in the majority of cases, AcCl) as a driving force for the condensation remained incompletely clear. Recently, this aspect has been studied in detail by Dmitriev and Ragulin [64,65,66]. Although acetyl chloride was proven to give the highest yield (approximately 70% yield in the reaction of benzaldehyde, an alkyl carbamate and an alkyl acrylate or methacrylate-derived phosphorus component), other systems were almost equally efficient. These systems included acetic anhydride alone or with the addition of trifluoroacetic or *p*-toluenesulfonic acid, which gave rise to 55-65% effectiveness of the condensation [64,65]. More importantly, careful inspection of the byproducts allowed the isolation of a biscarbamate **46** (Scheme 13) and suggest its structure as the key intermediate of the process [65]. 

Indeed, pre-synthesized biscarbamates reacted with phosphorous species **44** to the same extent as both substrates separately under analogous conditions [66]. To this end, an Arbuzov-type reaction was postulated as an amidoalkylation mechanism responsible for the novel P-C bond formation in **47** (Scheme 13). According to the suggestion, formation of a reactive nucleophilic phosphite and electrophilic acyliminium ion species from the corresponding substrates is mediated by carboxylic acid chloride or anhydride (here, trifluoroacetic anhydride) [66].

**Scheme 14:**
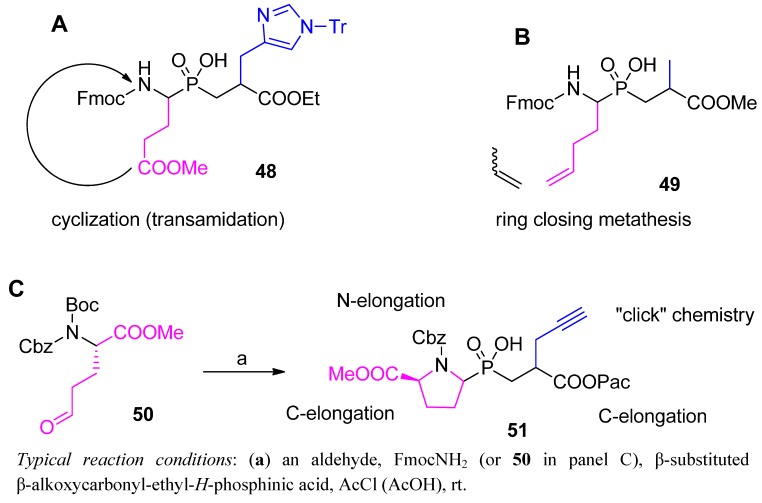
Examples of preparation of multifunctional phosphinic dipeptide analogues in the amidalkylation approach. Further modification points are highlighted.

Currently, the amidoalkylation approach to phosphinic pseudodipeptides seems to be considered advantageous over the phospha-Michael reaction. First of all, the scope of the amidoalkylation is broader, in particular with respect to the structural complexity of the P1 substituent (Scheme 14). 

P1 functionalities are well tolerated, as can be illustrated by effective preparation of a phosphinic compound bearing a glutamyl residue (**48**, Scheme 14A). Previous attempts to use phosphorus substrates incorporating additional carboxylate in an N → C silylation-activated process failed [23]. Alternative acetyl chloride-mediated amidoalkylation allowed the target molecule **48** to be obtained starting from complex substrates in one step and with a reasonable yield (48%) [50]. After incorporation into the target peptide structure (the analogue of thyrotropin-releasing hormone, cycloGlu-His-Pro-NH_2_), cyclization to the pseudoglutamyl residue occurred easily. Similarly, Huber *et al.* constructed a dipeptide that holds an unsaturated P1 side-chain (**49**, Scheme 14B, yield 64%) [67]. The properly protected building block was subsequently introduced into an octapeptide sequence. Ring closing metathesis involving another homoallylglycine residue produced a cyclic constrained inhibitor of β-secretase (BACE) with improved serum stability. Finally, a highly functionalized building block **51**, the precursor of P1 constrained peptidomimetics, is also shown. This building block was obtained in a cyclative manner from a suitably protected amino acid derivative bearing a distal side-chain aldehyde function **50** (Scheme 14C) [62]. Three points of reactivity of the product are designated for elongation of the structure in standard amide chemistry. The terminal alkyne group in the P1' position is a dipolarophilic site that offers further expansion to the heteroaromatic fragments.

#### 2.3.2. Phospha-Mannich and Kabachnik-Fields Reaction

**Scheme 15:**
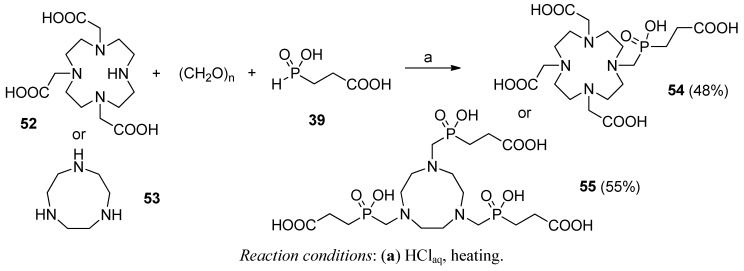
Synthesis of Glyψ[P(O)(OH)CH_2_]-Gly-substituted polyamines in a phospha-Mannich condensation with *H*-phosphinic acid and formaldehyde.

A related three-component condensation (phospha-Mannich reaction) was used by Hermann and co-workers to produce multifunctional complexing agents that hold a phosphinic glycyl-glycine unit. Starting from 2-carboxyethylphosphonous acid **39** (R = H) and a secondary polyamine, 1,4,7,10-tetraazacyclododecane-1,4,7-triacetate (**52**) or 1,4,7-trazacyclononane (**53**), phosphinate-functionalized products (**54** and **55**) were obtained (Scheme 15) [68,69]. As the condensation mechanism involves addition of the P-H function to the imminium ion formed *in situ* from the secondary amine and the carbonyl component, the process can be also considered as a variant of the Kabachnik-Fields reaction. 

The reaction proceeded with acidic catalysis and, although the reaction gave a moderate yield, this reaction was found to be simple and inexpensive. New ligands **54** and **55** are suitable for labeling biomolecules with transition metal radioisotopes in nuclear medicine [68,69,70,71,72]. For example, 1,4,7-trazacyclononane-based phosphinate has recently been described as an exceptionally convenient platform to develop gadolinium ^68^Ga(III) complexes as radiopharmaceuticals for positron emission tomography [72].

**Scheme 16:**
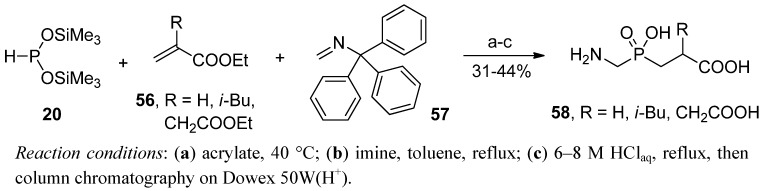
Synthesis of Glyψ[P(O)(OH)CH_2_]-Aa derivatives in subsequent addition reactions of an acrylate and *N*-trityl formimine to bis(trimethylsilyl) phosphonite.

A similar idea was earlier employed by Ragulin to obtain phosphinic dipeptides bearing glycine as the P1 fragment (**58**, Scheme 16) [63,73]. The whole synthesis was performed in a convenient manner involving a one-pot two-step sequential reaction of unsaturated compounds, first an acrylate **56**, then *N*-trityl formimine (**57**), with the silyl ester of hypophosphorous acid (**20**). The methodology for subsequent modification of the phosphorus component is complementary with one-pot modifications of the C → N approach (compare chapter 2.2), but the order of P-C bond formation is just reversed.

The overall yield of the reaction was diminished by 10–15% because of bis(2-carboxyethyl)-phosphinic acid formation. Appearance of the symmetrical byproduct can be suppressed entirely when at least a 5-fold excess of **20** to acrylate is used [29,30]. However, in such a case, an additional workup after the first stage would be demanded.

## 3. Modifications Following the C-P-C Formation

**Scheme 17:**
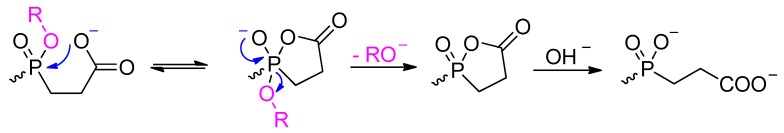
Suggested mechanism for a facilitated hydrolysis of a phosphinate ester under basic conditions. The reaction proceeds *via* a five-membered cyclic intermediate with neighboring-group assistance of β-carboxylate.

Formation of the Cbz-Aa_1_ψ[P(O)(OH)CH_2_]-Aa_2_-OAlk scaffold assembles a fundamental phosphinic structure that can rarely be used directly for further studies. At a minimum, the products of the synthesis need to be deprotected, which is achieved under standard conditions (for carbamates and esters). Alternatively, subsequent multidirectional transformations can be envisaged. These transformations typically concern two categories of changes: sequence propagation by amide bond formation at the N- and/or C-termini and/or structural modification of the R^1^/R^2^ substituent. Diverse chemistry has been applied for the latter purpose, including redox processes and synthesis of novel carbon-carbon and carbon-heteroatom bonds. All these reactions need careful orthogonal protection for at least three functionalities present in the molecule. Providing this protection is not an obvious task because these groups can behave unusually in this particular arrangement. The neighboring participation in the reactivity is best recognized for the pair phosphinate-β-carboxylate [74,75]. In the presence of the free carboxyl group, phosphinate protection is particularly labile, both under basic and acidic conditions. Such a hydrolysis is mediated by presumptive formation of a five-member cyclic phosphorus species (Scheme 17).

Other examples of cooperative effects were also reported [45,56,76]. One such example is cyclization of terminal groups. A free amino moiety and a methyl ester form the phosphinic analogue of diketopiperazine under basic conditions [45]. In this context, proper manipulations on these groups can be quite demanding. Achievement in this field is adequately illustrated by preparation of phosphinic dipeptides for solid-phase peptide synthesis.

### 3.1. Building Blocks for Solid-Phase Peptide Synthesis

**Scheme 18:**
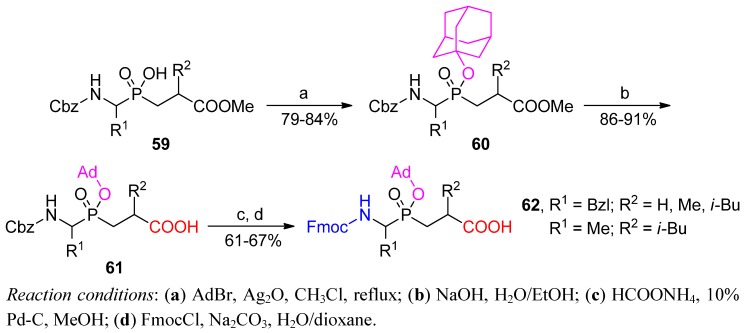
Preparation of phosphinic dipeptides as building blocks suitable for SPPS—the original procedure.

Application of phosphinic dipeptides as building blocks for peptide synthesis on a solid phase demanded the development of a convenient orthogonal protective group for the phosphinic moiety. After extensive studies in the Yiotakis group, the 1-adamantyl (Ad) group was found to be fully compatible with Fmoc methodology [16]. The blocking group was conveniently introduced in a silver oxide-mediated reaction (Scheme 18).

Typically, the whole procedure of Fmoc-Aa_1_ψ[P(O)(OAd)CH_2_]-Aa_2_-OH (**62**) preparation is a four-step protection/deprotection reaction sequence starting from Cbz-Aa_1_ψ[P(O)(OH)CH_2_]-Aa_2_-OR (**59**). The sequence consists of: (a) phosphinate adamantylation to obtain **60**; (b) C-terminal hydrolysis leading to acid **61**; (c and d) replacement of Cbz by Fmoc at the N-terminus. Cbz hydrogenolysis had to be carried out under particularly mild conditions to avoid cleaving the adamantyl group, so ammonium formate was applied as a hydrogen donor for a short-term catalytic process [16].

**Scheme 19:**
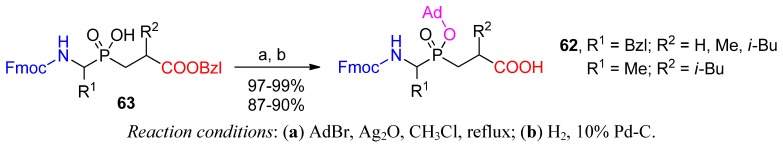
Final stages of phosphinic dipeptide preparation starting from N-Fmoc-α-amino-*H*-phosphinic substrates.

Since then, some modifications of the original approach have been developed. For example, the Fmoc protecting group is not sensitive to mild conditions of phosphinic acid activation (TMSCl/tertiary amine). Accordingly, Fmoc-AaPH could be applied in the Michael addition to synthesize **63** [77]. This reaction saved two manipulations at the N-terminus in the last stages of the procedure outlined in Scheme 18. However, to avoid basic conditions of alkyl carboxylate hydrolysis (also causing Fmoc cleavage), acrylate benzyl esters had to be applied (Scheme 19). Target compounds **62** were obtained with an excellent yield after adamantylation and hydrogenation.

**Scheme 20:**
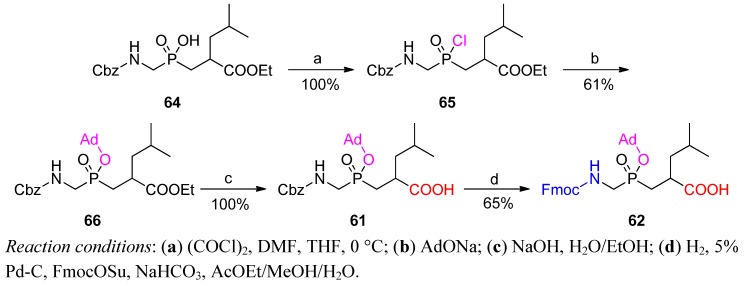
Modification of Fmoc-Aa_1_ψ[P(O)(OAd)CH_2_]-Aa_2_-OH preparation, performed for Gly-Leu analogue.

To avoid problems in a large scale and expensive silver-mediated adamantylation, Buchardt *et al.* suggested a two-step esterification that proceeded *via* the appropriate phosphinochloridate, and Buchardt *et al.* validated this approach for the Gly-Leu analogue (Scheme 20) [78]. Phosphinochloridate **65**, prepared quantitatively by oxalyl chloride reaction with substrate **64**, spontaneously reacted with sodium 1-adamantyl oxide to yield the P-protected compound **66**. *N*-Terminus protection and deprotection leading from **61** to **62** (R^1^ = H, R^2^ = *i*-Bu) were coalesced into one step by hydrogenolysis performed in the presence of the FmocOSu acylation agent.

**Scheme 21:**
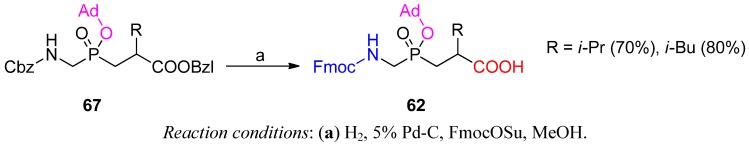
Transformation of Cbz-Glyψ[P(O)(OAd)CH_2_]-Aa_2_-OBzl into Fmoc-Glyψ[P(O)(OAd)CH_2_]-Aa_2_-OH in a single synthetic step.

Finally, all specific improvements were combined. Following the preparation of adamantylated products **67** (*via* the corresponding phosphinochloridates), all three remaining manipulations (tandem C-benzyl and N-Cbz removal together with simultaneous *N*-Fmoc introduction) proceeded in a single reduction step to give **62** (R^1^ = H, R^2^ = *i*-Pr or *i*-Bu, Scheme 21) [79].

**Scheme 22:**
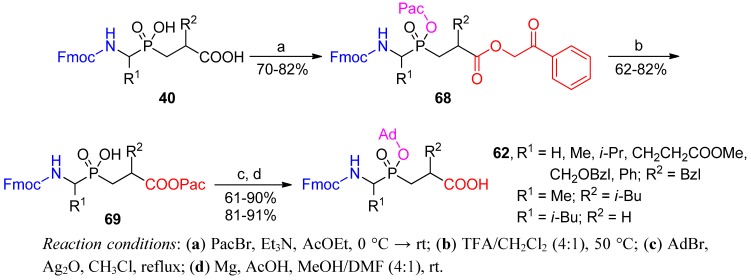
Chemoselective protection of a phosphinic dipeptide.

*N*-Fmoc-protected phosphinic pseudodipeptides **40** with both acidic functionalities free are easily accessible using a three-component condensation (compare Scheme 10) [61]. Having these pseudopeptides in hand, a unique chemoselective protection strategy was envisaged by Yiotakis and co-workers (Scheme 22) [76]. The phenacyl (Pac) group was installed on both the phosphinate and carboxylate of **68** under common reaction conditions. However, the action of a mild acid caused fully selective removal only from the hydroxyphosphinyl moiety (**69**). Such susceptibility to hydrolysis was tentatively explained by the assistance of the carbamate neighboring group, which might also participate in the formation of a five-membered reactive intermediate [76]. Two subsequent steps consisting of a typical adamantyl ester formation and Pac cleavage from the C-terminus completed the reaction sequence.

Fmoc-Aa_1_ψ[P(O)(OAd)CH_2_]-Aa_2_-OH building blocks (**62**) are commonly used in standard, automated Fmoc/benzotriazole solid-phase and combinatorial peptide synthesis [16,21,80,81,82,83]. Nevertheless, several reports consider P-protection unnecessary when particular short sequences are being targeted. The phosphinate functionality is poorly activated by standard coupling agents. The carboxylate can therefore be chemoselectively converted into the corresponding amide in the presence of the free phosphinic acid moiety, both in solution and on the solid phase by means of DCC (EDC)/HOBt, BOP, PyBOP, *i*-butyl chloroformate or other activators [32,36,61,84,85].

### 3.2. Side-Chain Substituents Modifications and Parallel Diversification

**Scheme 23:**
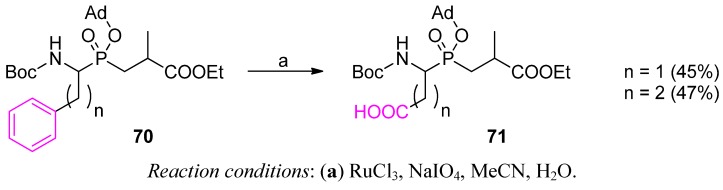
An oxidative approach to Aspψ[P(O)(O)CH_2_]-Ala and Gluψ[P(O)(O)CH_2_]-Ala phosphinic dipeptides.

Post-synthetic modifications of the phosphinate scaffold substituents concern mainly the C-terminal fragment of the molecule. One of the rare examples of the P1 transformation is an oxidative conversion of the phenyl ring of **70** into a carboxylate group. Mild conditions of oxidation allowed Georgiadis *et al.* to obtained Asp and Glu acidic residues in the Aa_1_ψ[P(O)(O)CH_2_]-Ala sequence (**71**) in moderate yield (Scheme 23) [23]. This problematic sequence is difficult to obtain in a typical phospha-Michael approach.

**Scheme 24:**
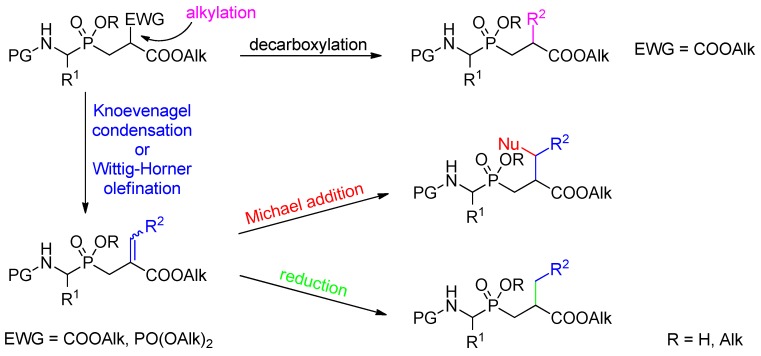
A general scheme of multidirectional modifications that have been envisaged starting from P1' active methylene phosphinic compounds.

Among chemical entities implemented in multidirectional and parallel P1' diversification chemistry, active methylene phosphinic synthons have been found to be one of the most interesting options (Scheme 24). 

Such synthons are easily prepared by addition of an alkyl methylidenemalonate or α-phosphonoacrylate to an α-amino-*H*-phosphinate. The Cα' proton acidity of the adduct can subsequently be utilized in several different ways. Two main routes involve alkylation of the malonate followed by decarboxylation of the product and a Wittig-Horner type of olefination (or, alternatively, Knoevenagel condensation) to introduce a double bond that can optionally be reduced or added with a nucleophile. Both methods have served to introduce a specific side-chain substituent to P1' portion of the molecule.

**Scheme 25:**
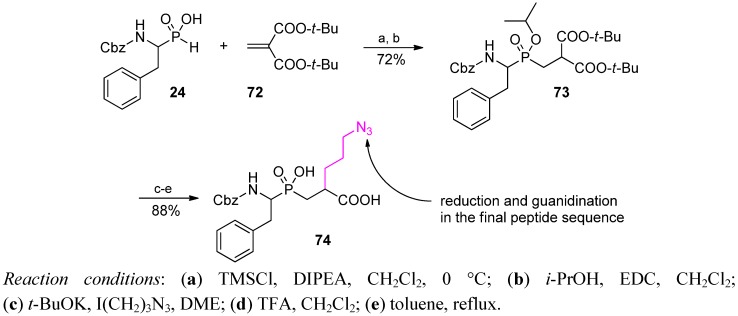
Construction of phosphinic dipeptide precursor of Pheψ[P(O)(OH)CH_2_]-Arg.

The alkylation strategy was used, for example, in the Ebetino group to prepare a precursor for the phosphinic Phe-Arg dipeptide isostere (**74**, Scheme 25) [44]. Di-*t*-butyl malonate-substituted pseudodipeptide **73**, obtained from **24** and malonate **72**, allowed effective alkylation with 1-azido-3-iodopropan (yield 88%). The building block was incorporated into the target peptide sequence and then subjected to one-pot modifications. The azido group was reduced to the amino group that finally was guanidinated *in situ*. 

**Scheme 26:**
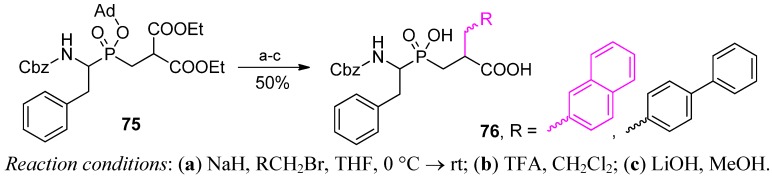
Alkylation of a malonate-derived phosphinic dipeptide.

Similarly, Matziari *et al.* introduced bulky arylmethyl P1' substituents to **75** using appropriate bromides and an alkylation approach (Scheme 26) [86]. The overall yield of products **76** was roughly 50%, outscoring the standard Michael-type addition that had previously been applied to provide the same 2-naphthyl compound (6.5%).

**Scheme 27:**
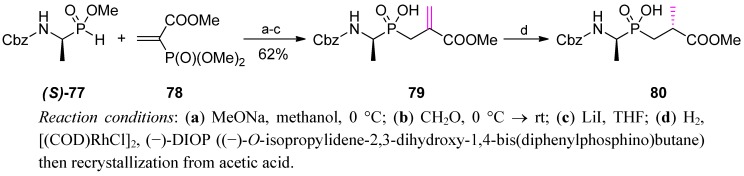
Synthesis of D-Alaψ[P(O)(OH)CH_2_]-D-Ala by a stereoselective reduction of dehydroalanine.

As an early example of the second variant (double bond formation and subsequent reduction, compare Scheme 24), Parsons *et al.* performed a stereoselective synthesis of D-Alaψ[P(O)(OH)CH_2_]-D-Ala inhibitor (**80**) of D-Ala-D-Ala ligase [31], a bacterial enzyme involved in cell wall biosynthesis. After addition of trimethyl phosphonoacrylate (**78**) to the *H*-phosphinic ester **77** and reaction with formaldehyde, stereoselective reduction of the pseudodehydroalanine compound **79** was employed (Scheme 27).

**Scheme 28:**
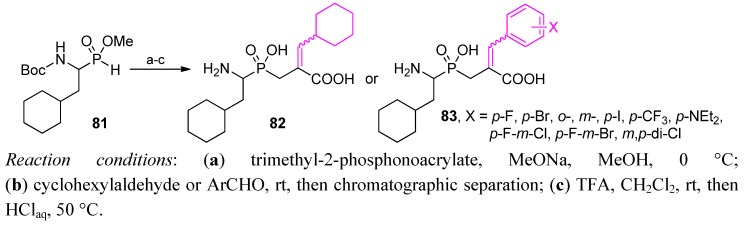
Synthesis of P1' dehydro phosphinic dipeptide inhibitors of renal dipeptidase.

More recently, an analogous approach was utilized to synthesize phosphinic dehydro analogues of cyclohexylalanine **81-**based inhibitors of renal dipeptidase (Scheme 28) [87,88]. After olefination of the phosphonoacetate with cyclohexylaldehyde or structurally diversified benzaldehydes, *Z* and *E* diastereoisomeric products **82** and **83** were separated chromatographically. *Z* isomers slightly predominated, typically in a 40%:30% ratio. Deprotected compounds, tested against the target peptidase, revealed the enzyme preference for the *Z* configuration (*IC*_50_ of a low nanomolar value).

**Scheme 29:**
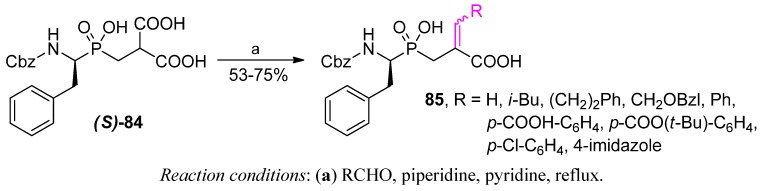
Synthesis of phosphinic pseudodipeptides containing P1' dehydroamino acid fragments by a Knoevenagel condensation.

To obtain P1' dehydroamino acid residues **85**, Matziari *et al.* employed a Knoevenagel-type condensation (Scheme 29) [86]. Different aldehydes reacted with phosphinic dipeptide-derived malonic acid ***(S)*-84** with a good yield and a preference for the *E* diastereoisomer up to 100%. The attempts at asymmetric reduction with rhodium catalysts and chiral ligands gave poor *de*.

**Scheme 30:**
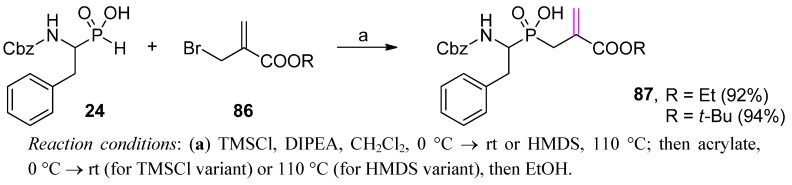
Reaction of α-amino-*H*-phosphinic acid with α-(bromomethyl)acrylates leading to dehydroalanine phosphinic dipeptides.

A mild, one-step and high-yielding conversion of P-H substrates, such as **24**, to P1' dehydroalanine phosphinic analogues **87** was also proposed in the Yiotakis group [89]. This conversion involved reaction of α-(bromomethyl)acrylic ester (**86**) with the trimethylsilylated phosphinate *via* a tandem Arbuzov reaction followed by allylic rearrangement (Scheme 30). The same results were obtained in the reaction of the unsaturated substrate with the acetoxy (instead of bromo) leaving group. A diversification potential for the dehydroalanine fragment was used to develop novel inhibitors of matrix metalloproteases (see below).

**Scheme 31:**
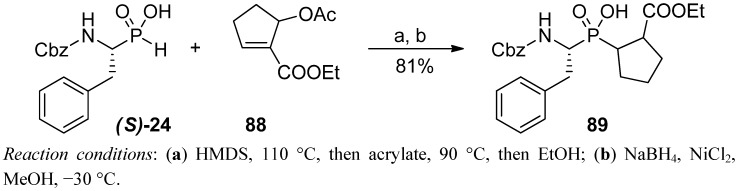
Preparation of a phosphinic dipeptide bearing the pseudoproline P1' fragment.

The tandem Arbuzov reaction (employing the acetoxy derivative **88**), combined with double bond reduction, was used to introduce the constrained P1' fragment of proline to the phosphinic dipeptide scaffold **89** (Scheme 31) [90]. Incorporated as a building block into a tripeptide sequence, it allowed to establish structural determinants of highly potent and selective inhibitors of the C-terminal domain of angiotensin-converting enzyme.

**Scheme 32:**
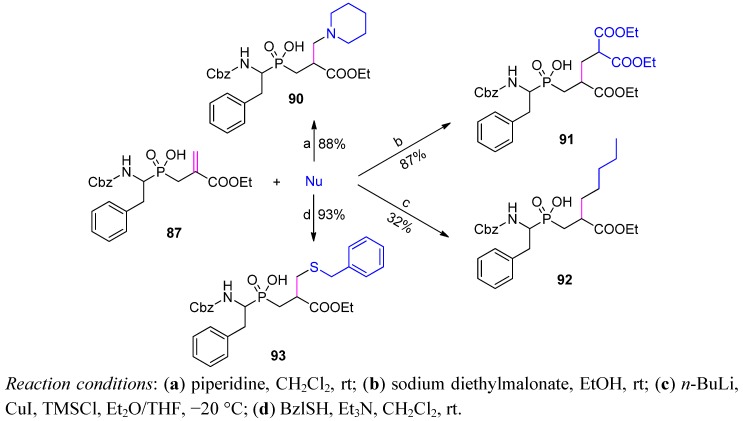
Addition of various nucleophiles (Nu) to P1' dehydroalanine phosphinic dipeptide.

Dehydroalanine synthon **87** can undergo a variety of subsequent transformations. As indicated, nitrogen, carbon and sulfur nucleophiles can easily be added to the activated double bond to yield products **90**-**93** (Scheme 32) [89]. The reactions were carried out under mild conditions and without the necessity of phosphinate group protection. The potential of P1' diversification in this manner was best recognized in the addition of thiols to acrylate implemented in a tripeptide sequence. The products were studied as inhibitors of matrix metalloproteases with particular attention to MMP-11 [91]. As a result, a diversity of tripeptide analogues of a general structure Cbz-(*L*)-Pheψ[P(O)(OH)CH_2_]-Cys(*R*)-(*L*)-Trp-NH_2_ were obtained and tested for complementarity to the S1' pocket of MMP-11. Remarkable selectivity of two orders of magnitude *versus* other members of MMP family was found for particular R substituents, for example, *o*-bromo or *o*-methoxyphenyl [91].

**Scheme 33:**
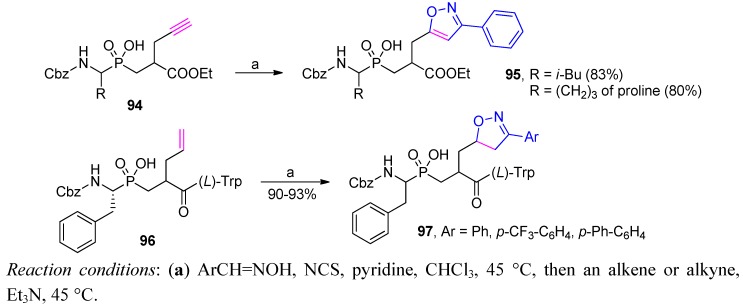
Synthesis of P1' isoxazole and isoxazoline derivatives of phosphinic peptide in 1,3-dipolar cycloaddition.

Other unsaturated groups present in the Cα' side-chain substituent of the phosphinic dipeptides can also be used for specific transformations. In particular, terminal alkenes and alkynes have been recognized in “click” chemistry to produce heterocyclic systems (Scheme 33) [40,47,48]. Both unsaturated systems (**94** and **96**) were successfully subjected to 1,3-dipolar cycloaddition as dipolarophilic substrates. The unsaturated systems reacted with nitrile oxides generated *in situ* from aryl oximes by oxidative chlorination. As a result, aryl-substituted isoxazoles or isoxazolines (**95** and **97**) were obtained with a good yield. The transformations were found to be equally effective in a dipeptidic building block and in an elongated peptide structure. Similarly to the previous example, the products were utilized to explore the specificity of the S1' binding pocket of selected metalloproteases, e.g., matrix metalloproteases, angiotensin- and endothelin-converting enzymes [40,47,48].

**Scheme 34:**
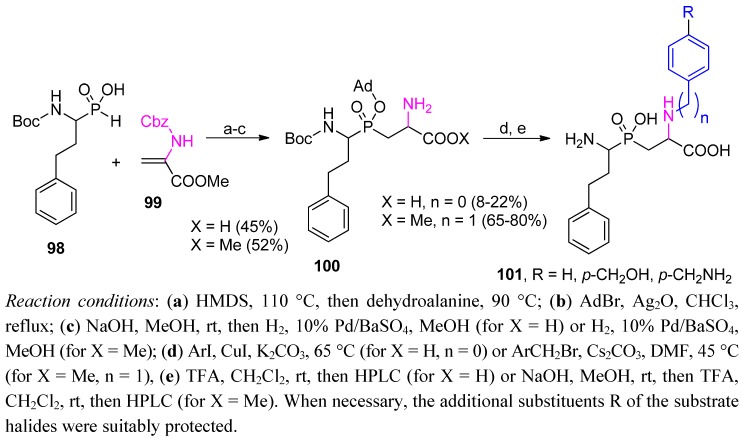
Parallel modification of phosphinic peptides containing a β' amino group in cross-coupling and alkylation reactions.

An expected anti-aminopeptidase inhibitory activity inspired elaboration of a phosphinic dipeptide building block containing a β' amino group that was suitable for parallel substitution [46]. According to the molecular modeling results, this group should be close to the position of the nitrogen atom in the transition state of the cleaved amide bond and, thus, favorably bound at the enzyme active site. The appropriate synthons were obtained by addition of the *H*-phosphinic analogue of homophenylalanine (**98**) to a dehydroalanine derivative **99** (Scheme 34). 

After typical protection/deprotection manipulations of the protecting group, two building blocks (**100**, X = H, n = 0 or X = Me, n = 1) were constructed and subjected to an cross-coupling with corresponding aryl iodides or an alkylation reaction with benzyl bromides [46]. Because of the structural complexity (namely, the presence of multiple functionality groups in both substrates), the yield of the cross-coupling reaction was rather low. The final products **101** needed to be purified on HPLC, which, in turn, allowed separation of diastereoisomers in the majority of cases.

## 4. Stereoselective Approaches

**Scheme 35:**
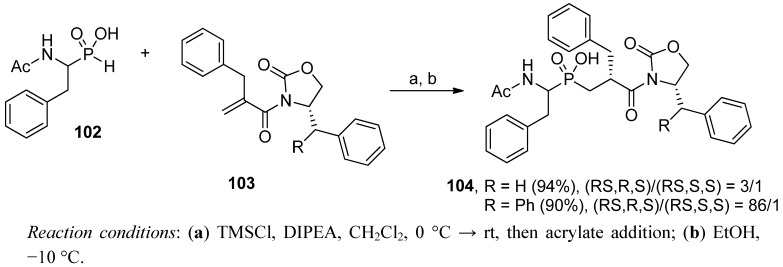
Diastereoselective addition of chiral oxazolidinone-derived acrylamides to α-amino-β-phenylethyl-*H*-phosphinic acid.

Enantiomerically pure α-amino-*H*-phosphinic acids are relatively readily available. *N*-Benzyloxycarbonyl-protected compounds form diastereomeric salts with commercially available chiral amines, e.g., 1-phenylethylamine. The salts crystallize smoothly to yield the required enantiomer [22]. The resolved phosphonous acids are not susceptible to racemization and tolerate diverse reaction conditions. Accordingly, optically pure α-amino-*H*-phosphinic acids are commonly used as the substrates in the phospha-Michael addition to produce P1 stereo-defined phosphinic pseudodipeptides. Even though the enantiomeric phosphonous acids rarely cause a significant induction of the newly appearing asymmetric center (Cα' atom), diastereomeric products can be separated simply by crystallization or chromatography [21,47]. The resolution is particularly effective when pseudodipeptides are included in a stereo-defined sequence of elongated peptides. In those cases, differentiation of even four diastereoisomers (epimeric on both Cα and Cα' atoms) on HPLC is not problematic [35,36]. Similarly, an astonishing difference in solubility in common solvents allowed separation of the four diastereoisomers of a phosphinic tripeptide by simple recrystallization [92]. With the aid of quantum mechanical calculations and molecular dynamics simulation these distinguishing physicochemical properties were attributed to the conformationally-specific pattern of inter- and intramolecular interactions among solute and solvent molecules. In accordance with this argument, stereoselective synthesis of phosphinic dipeptide analogues is not popular in the literature and concerns basically the P1' induction. Evans oxazolidinone-type auxiliaries were applied by the Ebetino group to induce the stereochemistry of the P1' position of Phe-Phe phosphinic dipeptide **104** (Scheme 35) [93]. 

To prepare chiral substrates **103**, α-benzylacrylic acid was coupled with the lithium salt of an oxazolidinone. Low-temperature trimethylsilyl-mediated addition of **102** to the acrylamides was found to produce an optimal induction. Quite naturally, top results of the *de* were achieved for the most crowded 4-diphenylmethyl-substituded oxazolidinone. Semiempirical AM1 energy calculation for the presumed enol ether intermediates pointed out that the *R* isomer is the favored product.

**Scheme 36:**
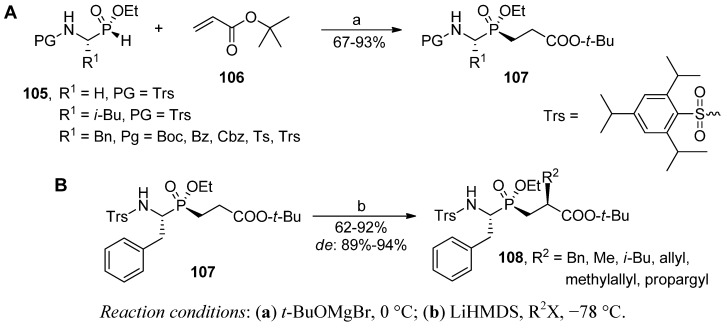
Diastereoselective phospha-Michael addition and Cα' alkylation of the resulting phosphinate dipeptide controlled by the defined phosphorus atom configuration.

A double diastereoselective approach to phosphinic dipeptide formation in a Michael addition followed by a Cα' alkylation was presented by Yamagishi *et al.* (Scheme 36) [94]. Using enantiomerically (R^1^ = H) or diastereomerically (R^1^ = *i*-Bu, Bn) pure P-chiral α-aminoalkyl-*H*-phosphinic acid esters (**105**) as substrates, the authors carried out addition of *t*-butyl acrylate (**106**) without a loss of the phosphorus chirality. The reaction, catalyzed efficiently by magnesium alkoxide, produced a single isomer **107** with an excellent chemical yield (Scheme 36A). The stereochemistry was controlled by the phosphorus atom configuration to give the complete retention for various P1 side chain substituents and N-protection groups.

Contrarily, the subsequent lithium-mediated alkylation step depended strongly on the steric parameters of the N-terminus. Excellent diastereoselectivity was observed for the most hindered Trs sulfonate protection (**107**, PG = Trs, R^1^ = Bzl), whereas induction was markedly poorer for other blocking groups [94]. Bulkiness of the side chain on the Cα position was not critical. Several different residues (alkyl, benzyl, alkenyl and alkynyl) were introduced in this way to the phosphinate dipeptide backbone as novel P1' substituents with a good yield (compounds **108**, Scheme 36B). Difficult N-deprotection can be considered as the single disadvantage of the method. The deprotection is a moderately effective three-step procedure including additional Cbz carbamoylation, Trs removal by SmI_2_ treatment and final Cbz hydrogenolysis [94].

**Scheme 37:**
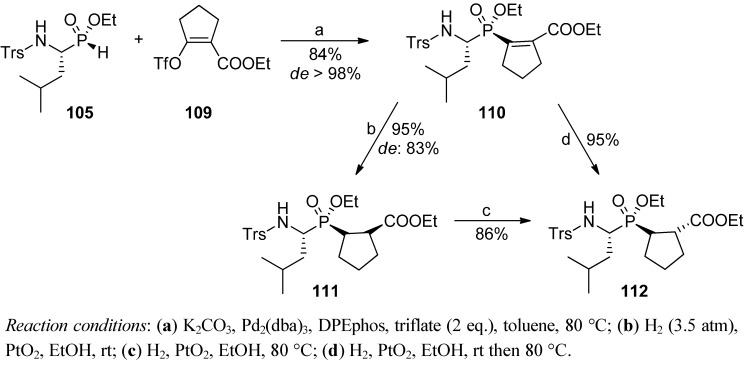
Diastereoselective cross-coupling and subsequent reduction leading to Leuψ[P(O)(OH)CH_2_]-Pro derivatives of the controlled pseudoproline configuration.

The addition of cyclopentenyl carboxylate to TrsLeuPH ethyl ester (**105**, R = *i*-Bu, PG = Trs) proceeded with difficulty. The methodology failed to prepare a Leuψ[P(O)(OH)CH_2_]-Pro derivative in the same manner. To overcome this drawback, a cross-coupling of the phosphorus substrate with the appropriate triflate **109** was envisaged (Scheme 37) [95]. After careful optimization of conditions, a palladium-catalyzed reaction carried out in the presence of K_2_CO_3_ as a base and DPEphos ((oxydi-2,1-phenylene)bis(diphenylphosphine)) as a chiral ligand yielded the unsaturated dipeptide **110** with a very good yield and an excellent *de*. Quite naturally, the subsequent reduction gave predominantly the *cis*-cyclopentane diastereoisomer **111** that could be isolated in a pure form after recrystallization or preparative TLC. Epimerization, occurring spontaneously to some extent during the hydrogenolysis, was preparatively induced by increase the temperature of reduction to yield the *trans*-cyclopentane configuration (**112**) [95].

**Scheme 38:**
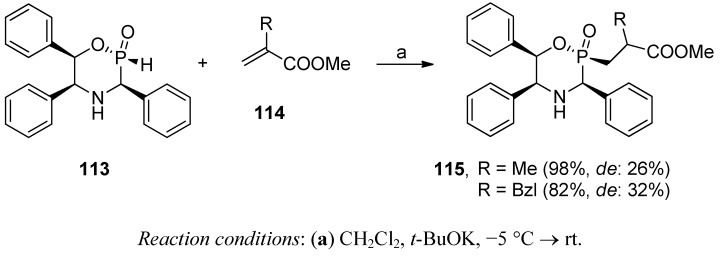
Diastereoselective addition of 2*H*-2-oxo-1,4,2-oxazaphosphinanes to acrylates.

Diastereoselective Michael-type addition of 2*H*-2-oxo-1,4,2-oxazaphosphinane **113** to olefins, including α,β-unsaturated esters (**114**, R = Me or Bzl, Scheme 38), was investigated by Monbrun *et al.* [96]. The reactions catalyzed by potassium *t*-butoxide proceeded with an excellent chemical yield and with complete retention of configuration of the phosphorus atom. 

The chiral induction of the newly formed stereogenic center in **115** depended strongly on the structure of the unsaturated substrates. For acrylates, the results were rather poor (~30%). More impressive data were indicated for substituents located closer to the sterically crowded heteroatom system.

In addition to the synthetic methods, stereoselective instrumental techniques were also applied to differentiate/resolve enantiomeric phosphinic dipeptide analogues. For example, an anion exchange chiral stationary phase based on cinchona alkaloids allowed resolution of dipeptide analogues in the reversed-phase HPLC mode [97]. The most effective *O*-9-(*t*-butylcarbamoyl)quinidine selector immobilized on a solid support was successfully utilized in chiral chromatography or electrochromatography separation of four stereoisomers of hPheψ[P(O)(OH)CH_2_]-Phe, a nanomolar inhibitor of leucine aminopeptidase [98,99].

## 5. Applications and Conclusions

The main field of application of phosphinic pseudopeptides concerns fundamental and practical aspects of inhibition of selected enzymes. This field of application mostly means representatives of two classes of catalytic proteins: ligases and hydrolases, in particular metallo-dependent proteases. Among others, these enzymes can catalyze, respectively, formation or cleavage of an amide bond in a metal ion-mediated process that proceeds *via* a tetrahedral transition state. C-P-C dipeptides comprise fundamental structural features of advantageous transition state analogue inhibitors. First, the dipeptides contain P1 and P1' fragments dedicated to explore the specificity of the corresponding S1 and S1' enzyme binding pockets. Second, the central phosphinate group is tetrahedrally-shaped and mimics the geometry and electron distribution of a diolate intermediate in the hydrolysis process. Third, phosphinic acid can be complexed to the central metal ion and block its catalytic function. Accordingly, phosphinic dipeptide isosters are broadly recognized as reversible, competitive inhibitors of many enzymatic targets [5,6,7,21].

**Scheme 39:**
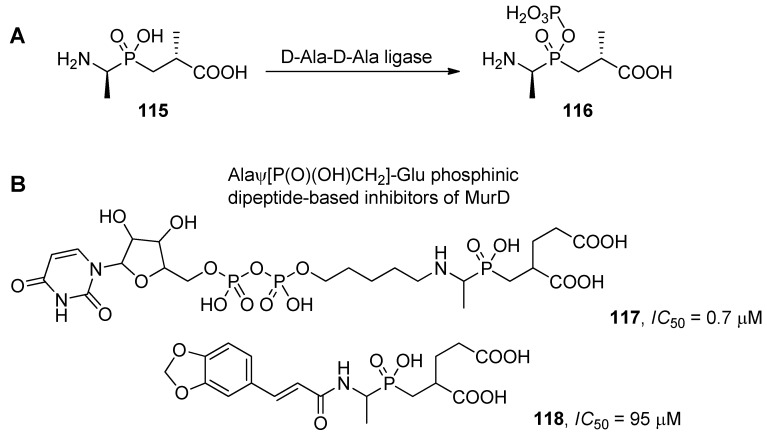
(**A**) The mode of action of D-Alaψ[P(O)(OH)CH_2_]-D-Ala, a phosphinic dipeptide inhibitor of D-Ala-D-Ala ligase; (**B**) Phosphinic dipeptide-derived inhibitors of MurD, another ligase involved in peptidoglycan biosynthesis.

Regulation of D-Ala-D-Ala ligase activity by D-Alaψ[P(O)(OH)CH_2_]-D-Ala (**115**) is one of the most prominent examples of ligase inhibition. The enzyme catalyzes condensation of two alanine molecules with ATP participation to form the terminal peptide of a peptidoglycan monomer. As the process is specific for bacteria, D-Ala-D-Ala ligase is an attractive antimicrobial target. The phosphinic analogue of the product of the reaction was found to be a micromolar inhibitor of the enzyme [31,100]. Interestingly, careful inspection of the mechanism of its action revealed that the phosphinate is not a typical transition state analogue. The phosphinate binds to the ligase in the phosphorylated form (**116**, Scheme 39A). Thus, the pseudodipeptide can be considered as a suicide substrate [100,101]. Identical phosphorylation of D-Alaψ[P(O)(OH)CH_2_]-D-Ala was discovered upon binding with VanA, a ligase responsible for natural resistance of vancomycin [102,103]. Other bacterial ligases involved in peptidoglycan biosynthesis have also been exploited as targets for antibacterial drug design using phosphinate dipeptide inhibitors [42,104,105,106]. Representative examples of UDP-*N*-acetylmuramoyl-L-alanyl:D-glutamate ligase (MurD) inhibitors (**117** and **118**) are shown in Scheme 39B. Recently, the L-Alaψ[P(O)(OH)CH_2_]-L-Phe ligand was also used for structural characterization of an L-amino acid ligase from *Bacillus subtilis* [107]. 

**Figure 3:**
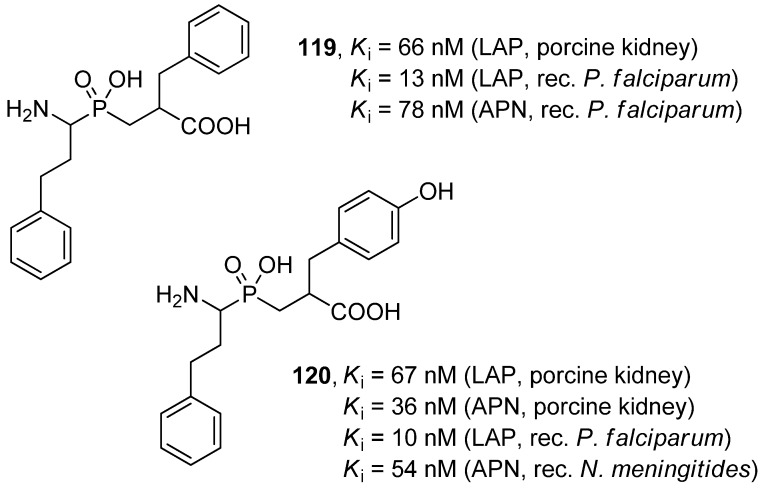
The structure and activity of hPheψ[P(O)(OH)CH_2_]-Phe and hPheψ[P(O)(OH)CH_2_]-Tyr phosphinic dipeptide inhibitors of leucine and alanyl aminopeptidases.

As far as metalloprotease targets are concerned, inhibition of leucine and alanyl aminopeptidases (LAP and APN) seems to be the most recognized example of biological activity of phosphinic dipeptide analogues [45,46,99,108,109,110,111]. LAP and APN are multifunctional broad-band specificity aminopeptidases involved in biological functions in both eukaryotic and prokaryotic cells. hPheψ[P(O)(OH)CH_2_]-Phe (**119**) and hPheψ[P(O)(OH)CH_2_]-Tyr (**120**), phosphinic compounds of an optimized structure, exhibited nanomolar activity towards mammalian (porcine kidney) [45], protozoan (recombinant *Plasmodium falciparum*) [112,113] and bacterial (recombinant *Neisseria meningitides*) [114] enzymes (Figure 3). 

In the most advanced studies, the phosphinates efficiently controlled the growth of *P. falciparum* cell lines and infection of a malaria model *in vivo* [112,113]. The structural basis of their activity was established by resolution of the crystal structures of the enzyme-ligand for both protozoan aminopeptidases complexed with the hPhe-Phe analogue [115,116]. The data altogether validated *P. falciparum* aminopeptidases as promising targets to treat malaria [113].

**Figure 4:**
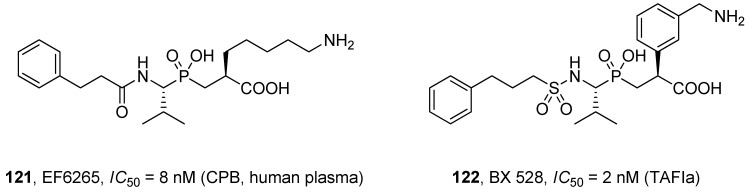
The structure and activity of phosphinic dipeptide inhibitors of TAFIa/carboxypeptidase B.

Plasma procarboxypeptidase B (thrombin-activatable fibrinolysis inhibitor, TAFIa) is a zinc-based exopeptidase proteolytically activated by thrombin into the active enzyme (carboxypeptidase B, CPB) that down-regulates fibrinolysis by removing the C-terminal lysine from fibrin fiber. Altering the action of CPB is a new way to target thrombosis-related diseases [117]. Phosphinic dipeptide analogues that comprise a lysine mimetic in the P1' position (**121** and **122**) were found to be excellent tools for this purpose. Compounds known in the literature as EF6265 and BX 528 (Figure 4) inhibited CPB with high affinity and excellent selectivity *versus* other carboxypeptidases and are prospective drugs for fibrinolytic therapy [118,119,120,121].

Other hydrolases, mostly zinc-containing, probed with phosphinic pseudodipeptide tools are listed below:

- angiotensin-converting enzyme 2, a mono-zinc carboxypeptidase, the first known human homologue of ACE [40];
- aminopeptidase A (glutamyl aminopeptidase) [122];
- NAALADase (*N*-acetylated-α-linked acidic dipeptidase), a metallo-dependent neuropeptidase [43];
- human renal dipeptidase and its bacterial homologue [87,88,123];
- M18 aspartyl aminopeptidase of *Plasmodium falciparum* [124];
- dinuclear zinc aminopeptidase PepV from *Lactobacillus delbrueckii* [125];
- D-Ala-D-Ala dipeptidase VanX, required for vancomycin resistance [126];
- chymotrypsin, cathepsin G and neutrophil elastase, serine proteases [127].

These examples do not complete the wide scope of phosphinic dipeptide utility. The majority of the currently-studied phosphinic compounds have used the fundamental building blocks for subsequent evolution into more complex systems. With respect to biological activity, the developed optimized sequences possess a refined potency/affinity to their targets that is also associated with an improved selectivity. Phosphinic fragments included in extended structures also acquire interesting metal complexing properties that have only occasionally been mentioned along the text. To conclude, phosphinic dipeptide preparation and diversification seem to be fundamental elements in a much broader and multifaceted area of phosphorus chemistry and biology.
